# Production of a single cyclic type of fructooligosaccharide structure by inulin‐degrading *Paenibacillus* sp. LX16 newly isolated from Jerusalem artichoke root

**DOI:** 10.1111/1751-7915.12358

**Published:** 2016-03-21

**Authors:** Zhihua Yao, Jiqiang Guo, Wenzhu Tang, Zhen Sun, Yingmin Hou, Xianzhen Li

**Affiliations:** ^1^ School of Biological EngineeringDalian Polytechnic UniversityDalian116034China

## Abstract

A novel inulin‐degrading bacterium was isolated from a soil sample collected on Jerusalem artichoke roots. It is a Gram‐positive, aerobic, motile and central endospore‐forming straight rod, and exhibits phenotypic properties being consistent with its classification in the genus *Paenibacillus*. The predominant cellular fatty acids were anteiso‐C15:0, C16:0 and anteiso‐C17:0. This strain represents a novel species of the genus *Paenibacillus* on the basis of phenotypic data together with phylogenetic analysis, and it is here designated as LX16 and deposited in China centre for type collection, China (= CCTCC 2015256). Strain LX16 could produce a cyclofructooligosaccharide fructanotransferase catalysing the formation of one type of fructooligosaccharide (FOS) from inulin. The FOS was identified as a cyclofructooligosaccharide with a degree of polymerization of 6. Such homology in inulin degradation products may be beneficial for the functional FOS production.

## Introduction

Fructooligosaccharide (FOS) is quickly emerging as an important food ingredient and pharmaceutical component because of its functional and nutritional properties (Sangeetha *et al*., 2005). It has a low caloric value and can successively improve the intestinal microflora by selectively stimulating resident bacteria such as bifidobacteria and lactobacilli, thereby making it difficult for pathogenic microbes to proliferate in the human gastrointestinal tract (Mussatto and Mancilha, 2007). Several beneficial aspects of FOS for human health include non‐cariogenicity, safety for diabetics, reduction in total serum cholesterol and lipid, relief of constipation and general human health improvement such as immune system activation and resistance to some infection (Respondek *et al*., 2011; Hunt *et al*., 2012; Silva *et al*., 2013). Some cyclic types of FOS have also been found to have potential capacities as novel host molecules in the medical, food and chemical fields because of the characteristic crown ether in the central part of the molecule and stabilizing effect on various materials in the freezing and thawing process (Uchiyama *et al*., 1993; Takai *et al*., 1994; Kanai *et al*., 1997).

Based on the preparation method, two types of FOS are commercially available. One type, referred to as GFn, is enzymatically produced from sucrose and comprises several fructose residues connected by β‐glycosidic linkages and a terminal sucrose residue at the reducing end (Hidaka *et al*., 1988). The other type of the commercial FOS, referred to as FFn, is produced by partially enzymatic hydrolysis of inulin (Kango and Jain, 2011). When FOS is produced by fructosyl transfer from pure sucrose by fructosyltransferase (EC 2.4.1.9) or β‐fructofuranosidase (EC 3.2.1.26) from bacterial and fungal sources, the maximal FOS production by a particular enzyme depends on its relative rate of transfructosylation and hydrolysis (Nguyen *et al*., 2005; Ghazi *et al*., 2007; Lafraya *et al*., 2011; Surin *et al*., 2012). The final composition of the product is a mixture of glucose, fructose, sucrose and FOS (Sangeetha *et al*., 2004). Therefore, a wider chemical spectrum of FOS is obtained, leading to difficulty in purification of FOS (Sangeetha *et al*., 2005; Guio *et al*., 2009).

Inulin occurs as a reserve carbohydrate in the root and tuber of plant such as the Jerusalem artichoke, and consists of a linear β‐2,1‐linked polyfructose chain displaying a terminal glucose unit (Chi *et al*., 2009). Inulin‐type FOS can be generated by the inulin hydrolysis with endoinulinases (EC 3.2.1.7) produced by several microbes, such as *Aspergillus ficuum*,* Kluyveromyces marxianus*,* Penicillium* sp., *Pseudomonas* sp., *Xanthomonas oryzae*,* Arthrobacter* sp. and *Bacillus smithii* (Roberfroid, 2007; Guio *et al*., 2009). However, the resulting products consist of a mixture of linear fructose oligomers with degrees of polymerization (DP) ranging from 2 to 10; additionally, some of these enzymes also produce glucose, fructose or sucrose as by‐products (Singh and Singh, 2010).

While searching for the potential microbial FOS producer in soil samples, we obtained an isolate that can selectively produce a dedicated/singly type of FOS structure from inulin. In this paper, we reported the isolation and partial characterization of this new inulin‐degrading strain, *Paenibacillus* sp. LX16, which is capable of degrading inulin into cyclofructooligosaccharide with a DP of 6 by using cyclofructooligosaccharide fructanotransferase (CFTase). To our knowledge, this is the first report on the conversion of inulin into a single FOS product by cultured microbes. The formation of one type of FOS molecules has strong potential for applications in producing functional oligosaccharides.

## Results

### Screening for inulin‐degrading microbes

When inulin‐degrading products were detected in the enriched inulin solution culture by thin‐layer chromatography (TLC) assay, a 10‐fold serial dilution was conducted, and single colonies were again cultured in the inulin medium. Several isolates were found to degrade inulin and to form oligosaccharide fractions in TLC plate. One strain showing the maximal oligosaccharide formation with little fructose/glucose production was obtained. The isolate was further streak‐purified on an inulin medium plate. One colony was designed as LX16 and used for further study.

### Phenotypic characteristics

Strain LX16 was Gram‐positive and motile by peritrichous flagella, with a straight rod shape. Each cell was 0.7–1.0 μm in diameter and 3.0–6.0 μm in length. The bacterium was an aerobe, and one endospore developed in the central region of the cell. The colonies on inulin agar plates were translucent and irregular, with a glistening and smooth surface. When spotted onto the centre of an inulin medium plate, LX16 cells swarmed out from the centre to form the branch patterns characteristic of tip‐splitting colony morphology.

The physiological and biochemical properties of the strain are shown in Table 1. Tests for catalase, oxidase and nitrate reductase were positive, and the test for urease was negative. Strain LX16 could not utilize citrate. The Voges–Proskauer test and the methyl red reaction were positive. Strain LX16 could digest gelatin but not starch, casein or pectin. The test for hydrogen sulfide production was negative, but the test for indole production was positive.

**Table 1:** Phenotypic characteristics of strain LX16

Characteristic	LX16	Characteristic	LX16
Gram staining	+	Hydrolysis of	
Straight rod	+	Gelatin	+
Endospore	+ (central)	Starch	−
Spore shape	Oval	Casein	−
Swollen sporangia	+	Pectin	−
Peritrichous flagella	+	H_2_S production	−
Anaerobic growth	−	M.R. reaction	+
Citrate utilization	−	V.P. test	+
Catalase	+	Nitrate reduction	+
Oxidase	+	Indole production	+
Urease	−	Growth in NaCl	
Litmus milk	+ (acid)	5%	+
Acid from glucose	+	7%	−

All the tested carbon sources supported the cell growth of strain LX16, they included inulin, fructose, sucrose, glucose and mannitol. Such nitrogen sources as peptone, tryptone, yeast extract, beef extract, urea and (NH_4_)_2_SO_4_ supported the cell growth of isolate LX16, but NH_4_Cl or NH_4_H_2_PO_4_ did not. The isolate grew at temperatures ranging from 5 to 37°C, but it grew optimally at 30°C. The isolate could grow over a pH range from 4.0 to 9.0, with the maximal cell mass obtained at pH 7.5.

### Cellular fatty acid

The major fatty acid composition of strain LX16 is shown in Table 2 together with those of closely related *Paenibacillus* species. The cellular fatty acid profile of strain LX16 was characterized by the presence of saturated fatty acids, such as anteiso‐C15:0 (55.0%), C16:0 (11.7%) and anteiso‐C17: 0 (10.3%), as the major fatty acids.

**Table 2:** Cellular fatty acid profiles (%) of strain LX16 and closely related *Paenibacillus* species

Fatty acid	1	2	3	4	5	6
Saturated fatty acids						
C14: 0	1.2	1.1	1.9	0.9	1.1	0.8
C15: 0	‐	2.6	1.7	2.7	0.3	0.7
C16: 0	11.7	6.3	10.2	9.7	15.6	11.2
C17: 0	‐	0.4	0.5	0.6	‐	‐
C18: 0	1.0	0.1	‐	‐	‐	‐
Unsaturated fatty acids						
C16: 1ω11c	1.3	‐	0.3	4.2	2.0	0.5
C 18: 1ω9c	3.8	‐	‐	‐	‐	‐
Branched fatty acids						
iso‐C14: 0	0.8	2.2	1.2	1.2	0.8	1.4
iso‐C15: 0	3.9	8.7	10.4	11.5	1.5	3.2
iso‐C16: 0	5.8	7.4	6.4	6.7	7.4	13.9
iso‐C17: 0	2.6	5.9	6.3	5.6	1.2	2.0
anteiso‐C15: 0	55.0	56.4	52.4	45.9	57.3	56.5
anteiso‐C17: 0	10.3	7.9	8.8	9.6	9.7	8.2

Strains: 1, LX16; 2, *Paenibacillus peoriae* (Lee and Yoon, 2008); 3, *P. kribbensis* (Lee and Yoon, 2008); 4, *P. lactis* (Scheldeman, 2004); 5, *P. lautus* (Scheldeman, 2004); 6, *P. glucanolyticus* (Scheldeman, 2004). Symbol: ‐, not detected.

### Phylogenetic analysis

To establish the phylogenetic position of the isolate LX16, its 16S rDNA gene was sequenced, and a 1461‐base sequence was obtained (GenBank accession no. KC581713). A preliminary comparative sequence search of the EMBL/GenBank database and Ribosomal Database Project‐II revealed that the 16S rDNA sequence of strain LX16 was most similar to that of species belonging to the genus *Paenibacillus* (Johnson *et al*., 2008; Cole *et al*., 2009).

**Figure 1:**
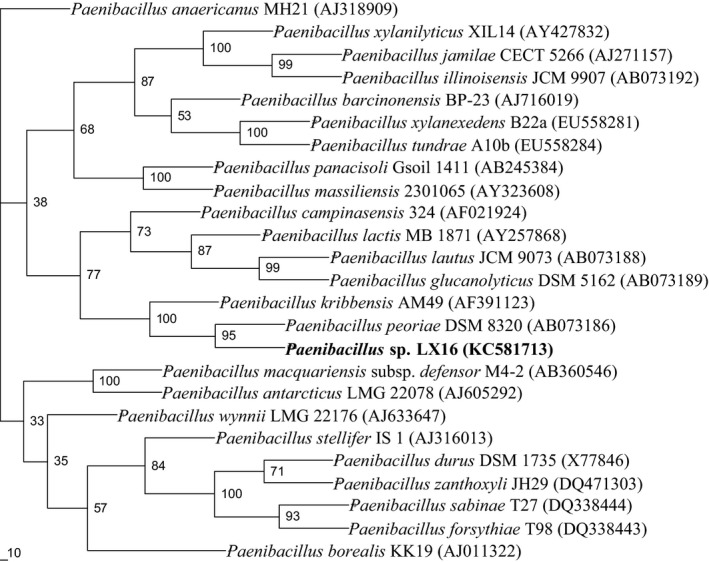
Neighbour‐joining phylogenetic tree showing the position of the LX16 isolate within the genus *Paenibacillus* based on 16S rDNA gene sequence data. GenBank accession numbers are shown in parentheses. Bootstrap values (expressed as a percentage of 100 replicates) are given at the branch points.

The similarity matrix derived from the sequences most similar to the 16S rDNA sequence of strain LX16 was calculated with the MegAlign program in the dnastar software package. The closest relative of strain LX16 was the type strain of *P. peoriae*, with a 16S rDNA sequence similarity of 99.6%. Strain LX16 also showed high sequence similarities with *P. kribbensis* (99.2%) and *P. glucanolyticus* (98.3%). The data set used for the construction of the phylogenetic tree contained 1398 base pairs in each sequence as a result of eliminating gaps and ambiguous nucleotides from the 16S rDNA sequences. The phylogenetic tree constructed by the neighbour‐joining method was shown in Fig. 1. Strain LX16 formed a phylogenetic cluster with *P. peoriae*.

### Fructooligosaccharide production by strain LX16

**Figure 2:**
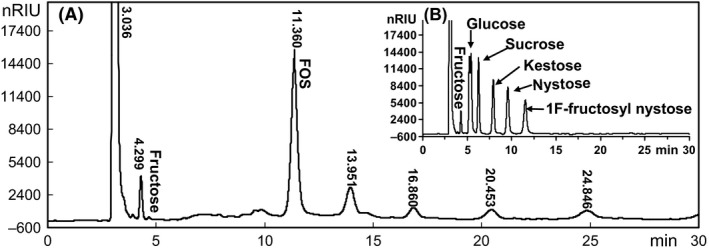
Chromatogram of inulin degradation products in the culture supernatant of strain LX16 after culture in inulin medium at 30°C for 2 days (A). In the sugar standards (B), the retention times correspond to fructose (4.293 min), glucose (5.431 min), sucrose (6.273 min), 1‐kestose (7.931 min), nystose (9.577 min) and 1F‐fructosyl nystose (11.541 min).

After cultivation of strain LX16 in the medium containing inulin as a carbon source, the inulin degradation products in the culture supernatant were analysed by high‐performance liquid chromatography (HPLC). As shown in Fig. 2, FOS was the predominant products in the culture supernatant of strain LX16 after 2 days of culture in the inulin medium. There was no detectable glucose or sucrose in the culture supernatant except a trace of fructose. The retention time of the product from inulin degradation by strain LX16 is close to that of fructosyl nystose (GF4), suggesting that the DP of product was possibly about 5.

### Fructooligosaccharide identification

**Figure 3:**
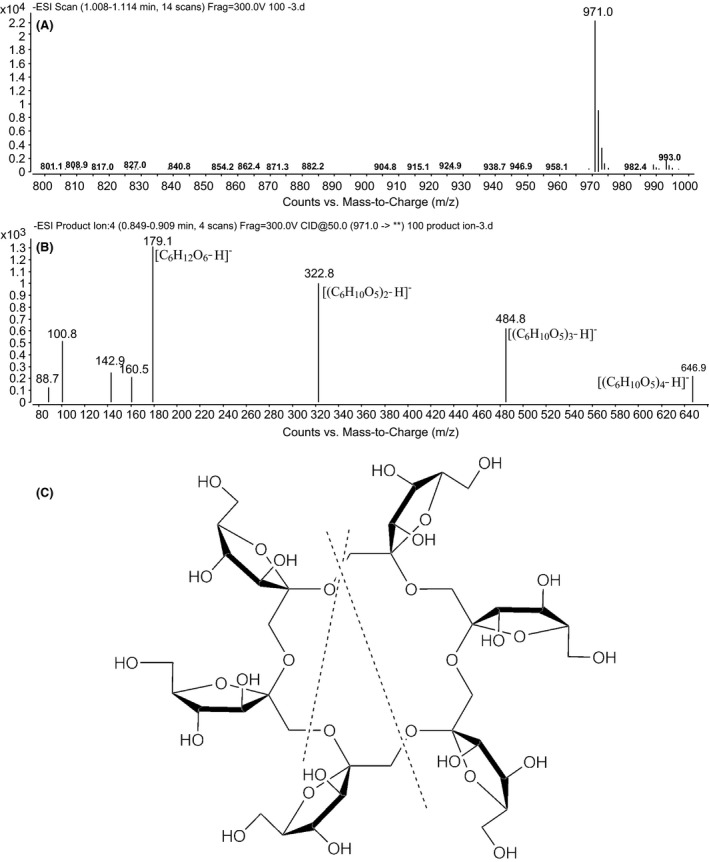
ESI‐MS analysis of the inulin degradation products. (A) Electrospray ionization spectra of the parent FOS; (B) electrospray ionization spectra of the fragmented FOS; (C) ways in which the FOS could be fragmented.

The inulin degradation product from the culture supernatant of *Paenibacillus* sp. LX16 was analysed by electrospray ionization mass spectrometry (ESI‐MS) in negative ion mode (Fig. 3). The molecular weight of the inulin degradation product was determined to be 972, as calculated from the m/z 971 ion corresponding to the deprotonated ion [G‐H]^−^ in the negative mode of ESI‐MS (Fig. 3A). The ions at m/z 179 (F), m/z 323 (F2), m/z 485 (F3) and m/z 647 (F4) were also observed when the ion at m/z 971 (F6) were further selected for MS2 fragmentation (Fig. 3B). The FOS structure and the presumed way to fragment FOS are shown in Fig. 3C.

### Production of cyclofructooligosaccharide fructanotransferase

**Figure 4:**
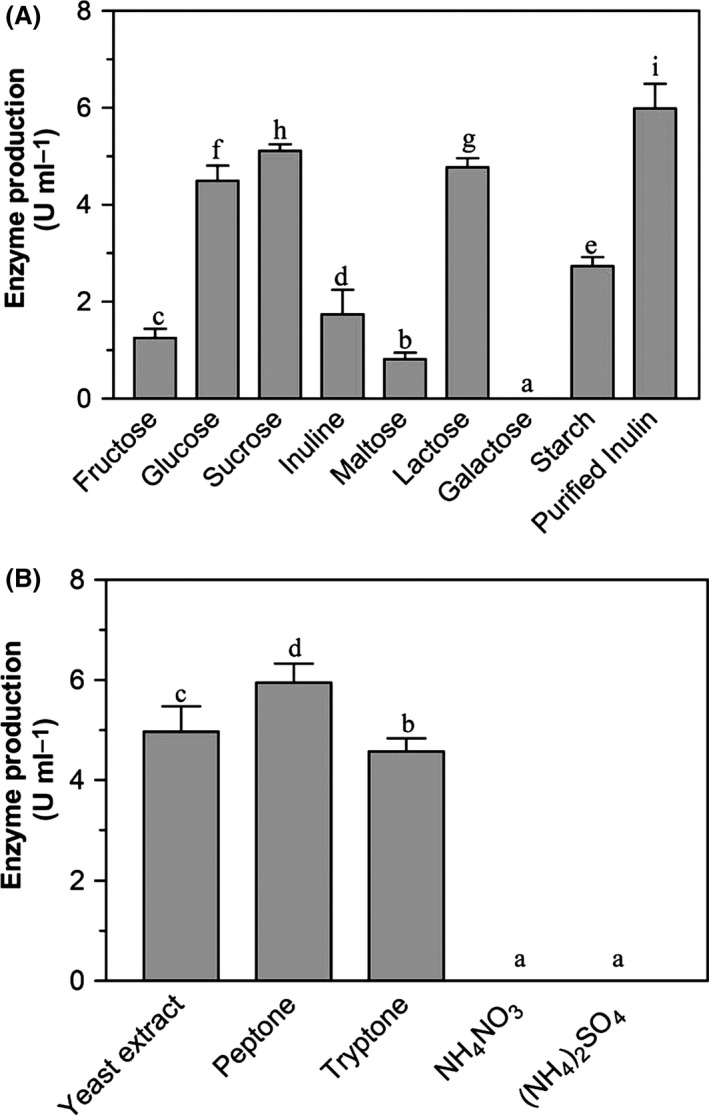
Cyclofructooligosaccharide fructanotransferase production after culturing *Paenibacillus* sp. LX16 in media with different carbon sources (A) and nitrogen sources (B) at 30°C and 160 r.p.m. Different letters at top of bars indicate significant differences at *P* < 0.05.

Cyclofructooligosaccharide fructanotransferase activity was detected in the culture supernatant of strain LX16 after incubation for 48 h in media with different carbon sources. As shown in Fig. 4A, enzymatic activity could be detected in cultures supplemented with all the tested carbohydrates except for galactose; the maximal activity was detected when the purified inulin was used as carbon source. Enzyme activity could be detected when organic nitrogen sources were used but not when inorganic nitrogen sources were used (Fig. 4B).

### Purification and characterization of cyclofructooligosaccharide fructanotransferase

**Figure 5:**
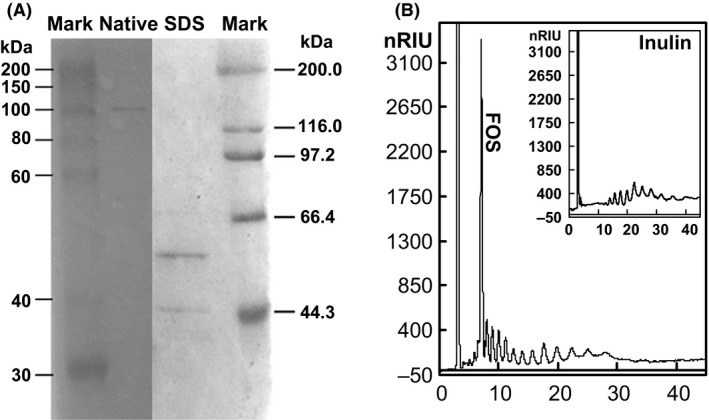
PAGE analysis of the cyclofructooligosaccharide fructanotransferase by silver staining in both native PAGE and SDS/PAGE (A), and the HPLC analysis of the inulin‐degrading products by the purified cyclofructooligosaccharide fructanotransferase (B).

Purification of CFTase was performed from the cell‐free culture of strain LX16 with a specific activity of 2.4 U mg^−1^, and the results were summarized in Table 3. The preparation gave a total protein recovery of 2.6% in a purification fold of 13.9 and the final product had a specific activity of 33.3 U mg^−1^. The purified CFTase was analysed by both native PAGE and SDS/PAGE stained with silver staining (Fig. 5A). One single protein band with molecular weight of 101 kDa was observed on the native PAGE, but two bands with molecular weight of 56.2 kDa and 44.8 kDa were observed on the SDS/PAGE. When the enzymatic degradation products of inulin by the purified CFTase were assessed by HPLC (Fig. 5B), one single type of FOS molecule was detected in the process of inulin degradation. The optimum temperature and pH for CFTase activity was 45°C and 7.0 respectively. The CFTase was active at pH ranged from 5.5 to 9.5, and at temperatures up to 55°C.

**Table 3:** Purification of cyclofructooligosaccharide fructanotransferase from the culture of *Paenibacillus* sp. LX16

Purification steps	Total activity (U)	Total protein (mg)	Specific activity (U mg^−1^)	Yield (%)	Purification (fold)
Cell‐free culture	610	250.1	2.4	100	1
(NH_4_)_2_S)_4_ precipitation	33.8	5.6	6.0	5.5	2.5
Phenyl Sepharose	19.5	0.86	22.7	3.2	9.5
DEAE‐Sepharose	15.8	0.47	33.4	2.6	13.9

## Discussion

The isolate LX16 was consistent with the typical characteristics of the genus *Paenibacillus* in the taxonomic properties and the cellular fatty acid profile was also similar to the pattern of the genus *Paenibacillus* (Shida *et al*., 1997). Therefore, based on the phenotypic analysis, strain LX16 should belong to the genus *Paenibacillus*.

Phylogenetic analysis based on 16S rDNA sequence further supported the affiliation of the newly isolated bacterium with the genus *Paenibacillus*. As shown in the neighbour‐joining phylogenetic tree (Fig. 1), strain LX16 clearly clustered into the clade of the genus *Paenibacillus*. In the tree, all the sequences derived from species in the genus *Paenibacillus* were more than 95% similar to that of strain LX16, except from the species *P. macquariensis* and *P. antarcticus*, which showed 94% similarity. The most closely related species was *P. peoriae*, with 99.6% similarity. These data were in accord with the proposal of using 95% 16S rDNA sequence similarity as a cut‐off value for delineating genera (Wagner‐Döbler *et al*., 2004). Therefore, the isolate LX16 should be classified as the member of the genus *Paenibacillus*.

As shown in Table 4, strain LX16 could be distinguished from its nearest phylogenetically neighbouring *Paenibacillus* species by some phenotypic differences, including positive tests for nitrate reduction, gelatin hydrolysis and cell growth in 5% NaCl, negative tests for citrate utilization, casein hydrolysis and anaerobic growth and by the existence of C18:1ω9c and absence of C15:0 among its cellular fatty acids (Table 2).

**Table 4:** Characteristics useful for differentiating strain LX16 from closely related *Paenibacillus* spp

Characteristic	1	2	3	4	5	6
Nitrate reduction	+	V	+	V	−	+
Citrate utilization	−	−	−	−	−	+
Casein hydrolysis	−	+	+	−	V	+
Gelatin hydrolysis	+	−	+	−	−	+
Anaerobic growth	−	+	+	−	+	+
Optimum growth temperature (°C)	30	28–30	30–37	30–40	28–30	NT
Growth with 5% NaCl	+	−	−	NT	+	+

Strains: 1, LX16; 2, *Paenibacillus peoriae* (Heyndrickx *et al*., 1996); 3, *P. kribbensis* (Yoon *et al*., 2003); 4, *P. lactis* (Scheldeman, 2004); 5, *P. lautus* (Heyndrickx *et al*., 1996); 6, *P. glucanolyticus* (Alexander and Priest, 1989). Symbols: NT, not tested; +, positive; −, negative; V, variable.

On the basis of the phylogenetic analysis, cellular fatty acid profile and phenotypic characteristics, it is apparent that strain LX16 cannot be assigned to any previously recognized species of the genus *Paenibacillus*. Therefore, the isolate LX16 should be a new member of the genus *Paenibacillus*, although the information on DNA–DNA hybridization is required to ascertain the final classification with certainty.

The ESI‐MS analysis of the inulin degradation product showed that parental ions from FOS were at m/z 971 but not at m/z 989 (Fig. 3A). Such ions corresponded to [(C_6_H_10_O_5_)_6_‐H]^−^. Moreover, there were no reducing terminals detected in the pure inulin degradation product. Thus, it may be concluded that this polymer of fructose must be cyclic. To confirm its cyclic structure, the ions at m/z 971 were further selected for MS2 fragmentation, and the ions of the fructose monomer were detected as the most abundant ions at m/z 179 by using an optimized collision energy (Fig. 3B). In addition, the ions at m/z 323, m/z 485 and m/z 647, corresponding to [(C_6_H_10_O_5_)_2_‐H]^−^, [(C_6_H_10_O_5_)_3_‐H]^−^ and [(C_6_H_10_O_5_)_4_‐H]^−^, respectively, were also observed in the ESI‐MS2 spectrum. These ions may be derived from the cyclic fructose polymer by fragmentation along the dashed lines shown in Fig. 3C. Therefore, the product derived from inulin by *Paenibacillus* sp. LX16 is a cyclofructooligosaccharide with a DP of 6; its structure is shown in Fig. 3C. This compound seems different from that detected in the HPLC chromatogram, in which the retention time corresponding to the inulin degradation products is close to that to GF4. However, this discrepancy is clarified by the cyclo‐product leading to a decreased retention time.

The CFTase converts inulin into cyclooligosaccharides by intramolecular transfructosylation consisting of six to eight β‐(2,1)‐linked D‐fructofuranoses, i.e. cycloinulohexaose, cycloinuloheptaose and cycloinulooctaose (Jeon *et al*., 2002). Currently, three microorganisms, *Bacillus circulans* (Kawamura and Matsuda, 2008), *B. polymyxa* (Jeon *et al*., 2002) and *B. macerans* (Lee *et al*., 2004), have been found to produce such a CFTase. However, only one type of FOS with DP 6 besides small sugars like fructose and partially degraded inulin residues was detected in the culture supernatant of strain LX16 (Fig. 2), suggested that strain LX16 degraded inulin to form a single cyclic type of FOS structure in addition to small sugars. Actually, the CFTase activity was detectable in the culture supernatant of strain LX16. Testing enzyme production by strain LX16 in the media with different carbon sources showed that the inulin induced the maximal CFTase activity (Fig. 4). The CFTase was a dimer comprising two non‐identical subunits with molecular weight of 56.2 kDa and 44.8 kDa respectively (Fig. 5A), being different from a monomer structure of the reported CFTases (Kushibe *et al*., 1995; Kim and Choi, 2001; Jeon *et al*., 2002). When the purified CFTase was used for inulin degradation (Fig. 5B), the transfructosylation result revealed the formation of a single type of FOS molecule besides some small sugars that presumably are the inulin‐degrading residues. This suggested that the CFTase of strain LX16 was distinct from the reported enzymes, because those CFTases catalysed the formation of three cyclofructooligosaccharides (Kushibe *et al*., 1995; Jeon *et al*., 2002).

## Conclusions

A Gram‐positive, aerobic, endospore‐forming and rod‐shaped bacterium was isolated from the rhizosphere of the Jerusalem artichoke, which is able to convert inulin into FOSs. According to phenotypic and phylogenetic analyses and its cellular fatty acid composition, the strain LX16 isolate should be assigned to the genus *Paenibacillus* within a novel species. *Paenibacillus* sp. LX16 could produce a CFTase that catalyses the formation of cyclofructooligosaccharide product from inulin.

## Experimental procedures

### Isolation of an inulin‐degrading strain and culture conditions

A soil sample (1 g) collected from Jerusalem artichoke root in the campus of Dalian Polytechnic University, China was suspended in 100 ml of 20% inulin solution with 0.1% yeast extract and incubated at 30°C with shaking (150 r.p.m.) for 5 days. A 10 ml aliquot of the culture liquid was then transferred to 100 ml of the identical fresh inulin solution and further incubated at 30°C with shaking. Samples of the culture were removed at intervals for TLC detection. After FOS was detected in the culture supernatant by TLC, a 10‐fold serial dilution of the enriched culture was prepared with sterilized saline and spread on inulin medium plates. All plates were incubated at 30°C for 24 h. Colonies growing on the inulin medium plates were randomly selected for culture in the inulin medium; part of each single colony was transferred to a fresh inulin medium plate and cultured at 30°C as a temporary stock, while another part of the colony was inoculated into inulin medium and incubated at 30°C with shaking. Isolates capable of forming FOS were preliminarily identified by TLC, and the culture supernatant was further analysed by HPLC. An isolate that could fully degrade inulin and produce FOS was selected and streaked on an inulin medium plate to ensure strain purity. A single arising colony was then used for further study.

Inulin medium contained 20 g inulin and 2 g yeast extract in 1000 ml of mineral solution. For solid media, 20 g agar was added per litre. The mineral solution contained (per litre) 0.05 g MgSO_4_·H_2_O, 0.7 g KNO_3_, 0.5 g K_2_HPO_4_ and 0.8 g NaCl at pH 6.2–6.4. The basal medium contained 0.05 g yeast extract in 100 ml of the mineral solution.

### Physiological and biochemical properties

As described previously (Murray *et al*., 1994), Gram staining was performed using the Hucker staining method, endospores were detected by the Schaeffer–Fulton staining method, and motility was observed in a hanging‐drop mount. A catalase assay was performed by detecting bubble formation when 3% (w/v) hydrogen peroxide solution was pipetted onto a colony after incubation on an inulin medium plate for 18–48 h. Oxidase activity was determined using 1% (w/v) tetramethyl‐p‐phenylene‐diamine as the substrate; the appearance of a purple reaction product within 30 s was considered a positive result (Smibert and Krieg, 1994). Urease activity was detected on Christensen urea agar slants by the presence of a red‐violet colour (Smibert and Krieg, 1994). Further tests, including indole production (method 2), methyl red reaction, Voges–Proskauer reaction, hydrogen sulfide production (method 2), nitrate reduction, citrate utilization (method 1), gelatin liquefaction (method 1) and hydrolysis of starch, pectin and casein were conducted using methods previously described (Smibert and Krieg, 1994). Growth ability on different substrates was detected by incubating isolate LX16 in basal medium supplemented with 0.2% (w/v) of each carbohydrate at 30°C for 7 days. Resistance to sodium chloride was observed by growing the strain on inulin medium supplemented with NaCl ranging from 1% to 7% (w/v).

The temperature range for cell growth was investigated by incubating isolate LX16 on inulin medium plates at different temperatures (5–80°C). The pH range for cell growth was evaluated by culturing the isolate in inulin medium at pH 2.0–10.0 for 48 h. The temperature and pH for maximal cell growth was determined by comparing the turbidity (OD_600_) of cultures after growth for 48 h. In carbon source and nitrogen source utilization tests, the OD_600_ of a liquid culture after cultivation in each medium was compared with that in the basal medium.

### 16S rDNA sequence analysis

Amplification and sequencing of the 16S rDNA gene was conducted by TaKaRa Bio. (Dalian, China). To avoid misreads as a result of PCR error, sequencing of each PCR fragment was repeated at least twice.

### Phylogenetic analysis

The closest known relatives of the isolate LX16 were identified by performing sequence searches of the GenBank/EMBL database using BLAST (Johnson *et al*., 2008) and by utilizing the Ribosomal Database Project‐II (Cole *et al*., 2009). The 16S rDNA sequences of the closest relatives were retrieved from the database, aligned using CLUSTALX (Thompson *et al*., 1997) and corrected manually. Only unambiguously aligned positions were used for phylogenetic analysis. Distance matrices were produced with the dnadist program of the phylip package software (Felsenstein, 2005), and an unrooted phylogenetic tree was constructed using the neighbor program contained in the phylip software package (V3.6) (Felsenstein, 2005). The statistical significance of the obtained groups was assessed by bootstrapping (100 replicates) using the programs seqboot, dnadist, neighbor and consense in the phylip software package. The percentage similarities between the 16S rDNA sequence of strain LX16 and other closely related bacteria were calculated using the MegAlign program in the dnastar software package (DNASTAR, Madison, WI, USA).

### Analysis of cellular fatty acid

For cellular fatty acid analysis, the isolate LX16 was cultivated in inulin medium overnight, and the cellular fatty acid composition was determined using the Sherlock Identification System (Tighe *et al*., 2000).

### Chemical analysis

Thin‐layer chromatography was used for monitoring inulin hydrolysis. A 0.5 μl aliquot of culture supernatant was spotted onto pre‐coated TLC plates (silica gel on polyester; Aldrich, St. Louis, USA) and developed. The developing solvent system was butanol–ethanol–water (5:3:2 by volume). The sugars were visualized by incubation with sulfuric acid–ethanol (1:9 by volume) at 105°C for 5 min. Sucrose, glucose (G), fructose (F; Sinopharm, Tianjin, China), 1‐kestose (GF_2_), nystose (GF_3_) and 1F‐fructosyl nystose (GF_4_) (Wakao Industry, Osaka, Japan) were used as standards.

Fructooligosaccharide analysis was also conducted using an Agilent 1260 series HPLC system with a refractive index detector and a Waters Spherisorb NH_2_ (250 × 4.6 mm) column. The injection volume was 10 μl, the mobile phase was acetonitrile‐water (70:30 by volume), the column temperature was 30°C and the detector temperature was 35°C (Silva *et al*., 2013).

The purified inulin degradation product was analysed by ESI‐MS with an Agilent 6400 Series triple quad LC/MS (Agilent, Santa Clara, USA). The mass spectrometer was operated in the negative mode. LC separation was performed in the isocratic mode with a water:acetonitrile ratio of 30:70. The injection volume was 10 μl, and the flow rate was set to 0.3 ml min^−1^. The scan range of the full mass scan was set from m/z 800 to m/z 1000. The MS parameters were set as follows: collision voltage 300 V; vaporization temperature 350°C; gas rate 8 l min^−1^; vaporization pressure 35 psi; capillary voltage 4000 V. The MS2 was performed with the optimized collision energy of 50.

### Enzyme assay

Fructotransferase activity was determined as previously described with some modification (Cha *et al*., 2001). After 0.5 ml of inulin solution (2% in 40 mM phosphate buffer, pH 7.0) and 0.5 ml of culture supernatant or purified fructotransferase were mixed and incubated at 45°C for 1 h, the reaction was stopped by heating in boiling water for 10 min. The formation of FOS was detected by HPLC. One unit of enzyme activity was defined as the amount of enzyme that produced 1 μmol of FOS per minute under the assay conditions. The inulin used for the assay was purified by ethanol precipitation to remove any glucose, fructose and sucrose present in the inulin solution. Protein content was measured by the method of Bradford (1976) using bovine serum albumin as a standard.

### Effect of carbon and nitrogen source on enzyme production

The strain LX16 was incubated in mineral solution with 0.05% yeast extract and 0.5% of each tested carbon source at 30°C and 160 r.p.m. for 48 h. Tested carbon sources included fructose, glucose, sucrose, inulin, lactose, galactose, maltose and starch. CFTase activity was determined. The influence of nitrogen source was assayed after strain LX16 was incubated at 30°C and 160 r.p.m. for 48 h in mineral solution with 0.5% inulin and 0.1% of different nitrogen sources, including yeast extract, peptone, tryptone, NH_4_NO_3_ and (NH_4_)_2_SO_4_.

### Production of inulin‐degrading product

An overnight culture of the isolate LX16 in inulin medium was inoculated into mineral solution with 10% inulin and 0.2% yeast extract and incubated at 30°C with shaking at 160 r.p.m. Samples of the culture sample were removed at various intervals, and inulin degradation was detected by TLC assay after centrifugation at 8000 *g* for 10 min. When the inulin was fully converted into FOS, the inulin‐degrading components in the culture supernatant were further analysed by HPLC and used for molecular characterization.

### Purification of inulin‐degrading product

After precipitation with ethanol, inulin‐degrading products were applied to a silica gel filtration column (0.9 by 20 cm) previously equilibrated with a solution of butanol–ethanol–distilled water (ratio of 5:3:2). The products were eluted at room temperature with the same solution. The eluted products were then detected by HPLC.

### Purification of cyclofructooligosaccharide fructanotransferase

The CFTase purification was performed as described by Yang *et al*. (2014). Briefly, ammonium sulfate precipitation was carried out by dual precipitation of 30% and 75% saturation; hydrophobic interaction chromatography was performed on a column of phenyl Sepharose FF by a linear gradient elution of 1–0 M (NH_4_)_2_SO_4_ in 20 mM phosphate buffer (pH 7.0); anion‐exchange chromatography was carried out on the column of DEAE‐Sepharose by linear gradient elution of 0–1 M NaCl in 20 mM phosphate buffer (pH 7.0). Active fractions were pooled and the purity of CFTase was assessed by native PAGE and SDS/PAGE respectively.

### Temperature and pH optima

For determination of the optimum temperature, the CFTase activity was assayed at different temperatures (25–60°C). The optimum pH was determined by measuring the CFTase activity in 20 mM phosphate buffer at pH 6.0–8.0.

### Statistical analysis

All of the assays and determinations described in this paper were performed in triplicate unless otherwise stated. The data were subjected to one‐way analysis of variance to detect statistical significance.

## Conflict of interest

None declared.
